# Total time of operation is a risk factor of stroke-associated pneumonia in acute ischemic stroke patients with intra-arterial treatment

**DOI:** 10.1097/MD.0000000000003958

**Published:** 2016-07-22

**Authors:** Rui Liu, Wei Li, Yaoyang Li, Yunfei Han, Minmin Ma, Wusheng Zhu, Min Li, Qiliang Dai, Yuezhou Cao, Gelin Xu, Xinfeng Liu

**Affiliations:** a Department of Neurology, Jinling Hospital, The Second Military Medical University; b Department of Neurology, Jinling Hospital, Southern Medical University, Nanjing, China.

**Keywords:** complication, mechanical thrombolysis, outcome, pneumonia, stroke

## Abstract

**Background and purpose:**

Stroke-associated pneumonia (SAP) is associated with poor functional outcome in patients with acute ischemic stroke (AIS). The objective of this study was to identify predictors of SAP in patients underwent intra-arterial treatment (IAT).

**Methods:**

Consecutive patients with AIS within 6 h from the symptom onset underwent IAT were enrolled. Independent predictors of in-hospital SAP after AIS were obtained using multivariable logistic regression. Kaplan–Meier survival curves were calculated and compared by the log-rank test.

**Results:**

Of 165 patients with AIS in the study period, 102 (61.8%) underwent IAT. Twenty-two (21.6%; 95% confidence interval [CI], 14.7–29.4) experienced SAP. Patients with SAP were older (69.2 vs 62.9 years, respectively; *P* = 0.011), more severely affected (National Institutes of Health Stroke Scale score, 18 vs 9, respectively; *P* = 0.004), more likely to underwent symptom of dysphagia (86.4% vs 15%, respectively; *P* < 0.001), lower Glasgow Coma Scale score (9 vs 13, respectively; *P* < 0.001), and longer operation time (149.5 vs 123, respectively; *P* < 0.001) than those without SAP. Only symptom of dysphagia (adjust odds ratio [OR], 12.051; 95% CI, 3.457–50.610; *P* < 0.001) and total time of operation (adjust OR, 1.040; 95% CI, 1.009–1.071; *P* < 0.001) were identified as independent predictors of SAP. Patients with SAP had stable or improved deficits after AIS with IAT (*P* < 0.001).

**Conclusions:**

Besides dysphagia, total time of operation is a risk factor of SAP in patients with AIS with IAT.

## 1 Introduction

Intra-arterial therapy (IAT), either through local delivery of thrombolytic agents or mechanical thrombectomy, has provided credible evidence for the treatment of acute ischemic stroke (AIS) within 6 h after symptom onset.^[1–3]^ Complications frequently complicate stroke and have a significant impact on prognosis, length of stay, and healthcare costs.^[4–6]^ Stroke-associated pneumonia (SAP) is the most common cause of mortality following acute stroke and is also associated with poor functional outcome.

Although the results of previous studies demonstrated the effectiveness of IAT, SAP may be still an important reason causing a higher totally cost and a relatively low proportion of patients had a modified Rankin score (mRS) of 0 to 2 at the 90-day follow-up assessment.^[1,7]^ Identifying patients with the highest risk of developing SAP may be helpful for treatment and prevention.

Traditionally, SAP is considered to be secondary to aspiration.^[8–10]^ Several risk factors for SAP have been identified, such as older age, male sex, diabetes mellitus, hypertension, atrial fibrillation, congestive heart failure, chronic obstructive pulmonary disease, pre-existing dependency, stroke severity, stroke subtypes, and dysphagia.^[11–16]^ Previous studies also showed that the incidence of SAP depends on the setting. It varies between 10% and 57% for patients treated in intensive care units and between 4% and 12% for patients treated exclusively in stroke units.^[17]^ Some studies have provided evidence for stroke-induced immunodepression.^[18,19]^

We conducted this study to determine how frequently patients with AIS underwent IAT get SAP, and identify predictors of SAP in patients underwent IAT.

## 2 Subjects and Methods

### 2.1 Study design

The study population was consisted of consecutive patients with AIS within 6 h from the symptom onset prospectively enrolled in the Nanjing Stroke Registry Program^[20]^ from March 2010 to July 2014. The study was approved by the institutional review boards at Jinling Hospital, and all participants or their surrogates provided written informed consent. The study was carried out in accordance with the Helsinki Declaration. The potential intra-arterial treatment (IAT) were evaluated based on computed tomographic (CT) angiography, magnetic resonance angiography, or digital-subtraction angiography, and a score of 2 or higher on the National Institutes of Health Stroke Scale (NIHSS) score in all patients. IAT includes arterial catheterization with delivery of a thrombolytic agent, mechanical thrombectomy, or both. Radiological and angiographic imaging was reviewed. We documented location of vascular occlusion, thrombolysis in cerebral infarction (TICI) flow pre and postprocedure, and procedural complications. Data were collected by the study physicians through review of medical records and neurological examination. Neurological status was evaluated on a daily basis. Pretreatment NIHSS and 90-day mRS were assessed by certified stroke.^[1,21]^

Exclusion criteria including: history of life-threatening allergy to contrast medium, not just rash; oral anticoagulant therapy with International Normalized Ratio > 3.0, known hemorrhagic diathesis, or coagulation factor deficiency; baseline platelet count < 30,000/μL; creatinine ≥ 3 mg/dL; illness with <1 year anticipated life expectancy.^[22]^

After intravenous thrombolysis within 36 h, the patient with any bleeding signs in CT scan and with a deterioration of 4 or more points in the NIHSS score should be diagnosed as symptomatic intracerebral hemorrhage.^[22,23]^

In this study, the medical diagnosis of pneumonia requires a new and persistent infiltration or solidifies presence on at least 1 chest x-ray, or at least 2 serial chest X-rays in cases of underlying lung disease. Some clinical signs should be combined, including fever, leukocytosis, or leukopenia, and mental status change who are older than 70 years. Two of the following signs should be added to above signs, including rales, cough, purulent sputum or sputum characteristics change, and gas exchange worsening.^[22]^

IAT efficacy was ultimately evaluated based on restored flow postprocedure, a TICI score of 2b or 3, and the absence of periprocedural complications.^[24]^ Good outcome was defied as mRS ≤ 2 at 90 days, and mortality was defied as mRS = 6 at 90 days. These parameters were acquired by telephone interview with patients, relatives, or family doctors.

### 2.2 Statistical analysis

The Statistical Product and Service Solutions (SPSS, IBM Corporation, Armonk, NY) program (version 22) was used to analyze the data. Depending on the distribution of data, continuous variables are expressed as means (standard deviation) and medians (interquartile ranges [IQRs]). Nominal and categorical variables and percentages for categorical variables are expressed as absolute numbers and percentages. The correlation between categorical variables was determined by Chi-squared test. A *t* test between continuous variables and a Mann–Whitney test between scores were performed. The odds ratios (ORs) were estimated by adjusted logistic regression. A *P* < 0.1 were entered into the logistic regression model. In order to study the ability of SAP for mortality prediction, we calculated Kaplan–Meier survival curves and compared by the log-rank test. Hazard ratios were reported. A *P* < 0.05 was considered as significant.^[22]^

## 3 Results

### 3.1 Patient characteristics

Of 165, 102 (61.8%) hospitalized patients with AIS underwent IAT in the study period. There were 36 women (35.3%) and 66 men (64.7%). The median interval from stroke onset to puncture was 253.6 min (range, 58–363 min). Mean age of the study patients was (64.2 ± 10.5) years (range, 40–85 years). The median NIHSS score was 15 (IQR, 10.0–19.3).

Before IAT, 28 patients (27.5%) were treated with intravenous alteplase. Six patients (5.9%) were given in no intervention. Actual intra-arterial therapy was performed in 96 of 102 patients (94.1%). Intra-arterial thrombolytic agents were given to 36 patients (35.3%). Retrievable stents were used in 65 patients (63.7%). Acute cervical carotid stenting used as a simultaneous second revascularization procedure was performed in 12 patients (11.8%).

### 3.2 Factors influencing SAP

**Figure 1:**
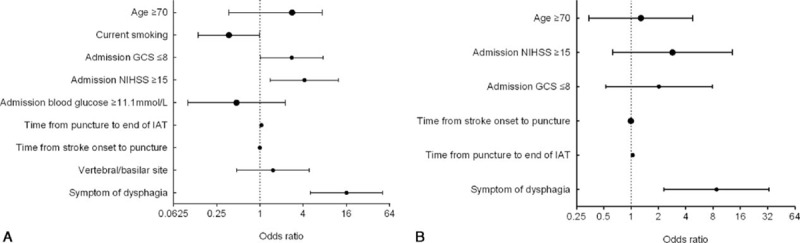
(A) Univariate analysis of parameters associated with in-hospital SAP after AIS; (B) multivariate analysis of parameters associated with in-hospital SAP after AIS. AIS = acute ischemic stroke, GCS = Glasgow Coma Scale, IAT = intra-arterial treatment, NIHSS = National Institutes of Health Stroke Scale, SAP = stroke-associated pneumonia.

**Figure 2:**
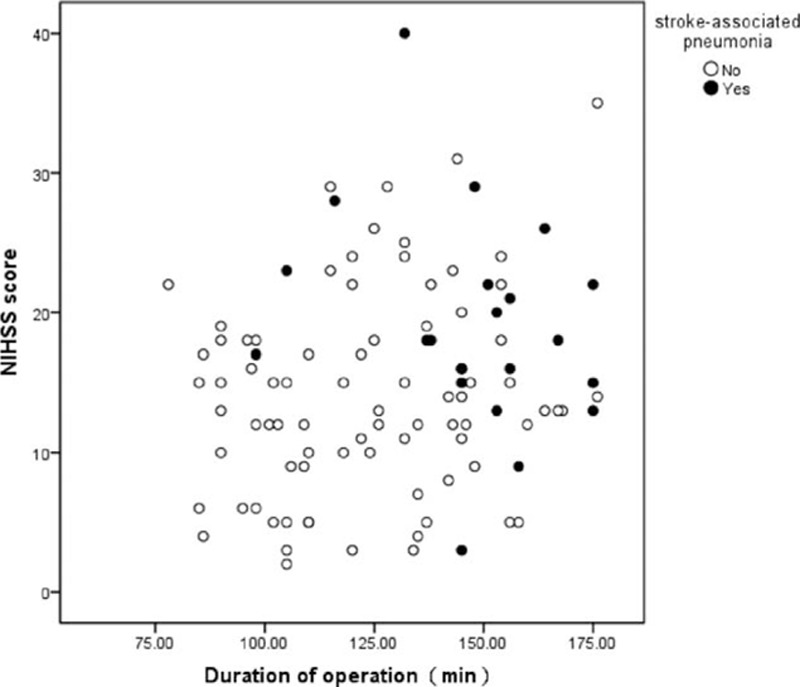
Scatter diagram of stroke-associated pneumonia distribution according NIHSS score and total time of operation. NIHSS = National Institutes of Health Stroke Scale.

Twenty-two intra-arterial treated patients (21.6%) were diagnosed as pneumonia during hospitalization (95% confidence interval [CI], 14.7–29.4). The average duration of hospital stays is 10.8 ± 9.0 days. The clinical symptom of patients with pneumonia (NIHSS score, 18) was more serious than those without pneumonia (NIHSS score, 9) (*P* = 0.004). In addition, patients with pneumonia were older (69.2 vs 62.9 years, respectively; *P* = 0.011). Symptom of dysphagia was occurred more (86.4% vs 15%, respectively; *P* < 0.001) and Glasgow Coma Scale (GCS) score was lower (9 vs 13, respectively; *P* < 0.001) in patients with SAP. The operation time of patients with SAP was longer (*P* < 0.001). No association was found between pneumonia and TOAST classification, and no association was found between pneumonia and responsible vessels (Table 1). In a univariate analysis, SAP was significantly influenced by 5 variables (Table 1): age ≥ 70 years (crude OR, 2.8; 95% CI, 1.1–7.4; *P* = 0.037), the severity of neurologic deficits (NIHSS score ≥ 15; crude OR, 4.156; 95% CI, 1.4–12.4; *P* = 0.010), admission GCS ≤ 8 (crude OR, 2.769; 95% CI, 1.0–7.6; *P* = 0.048), total time of operation (crude OR, 1.045; 95% CI, 1.020–1.071; *P* < 0.001), and symptom of dysphagia (crude OR, 16.029; 95% CI, 5.066–50.717; *P* < 0.001). The multiple logistic regression analysis showed only symptom of dysphagia (adjust OR, 12.051; 95% CI, 3.457–5.610; *P* < 0.001) and total time of operation (adjust OR, 1.040; 95% CI, 1.009–1.071; *P* = 0.011) were identified as independent predictors of SAP (Table 2, Figs. 1 and 2).

**Table 1:**
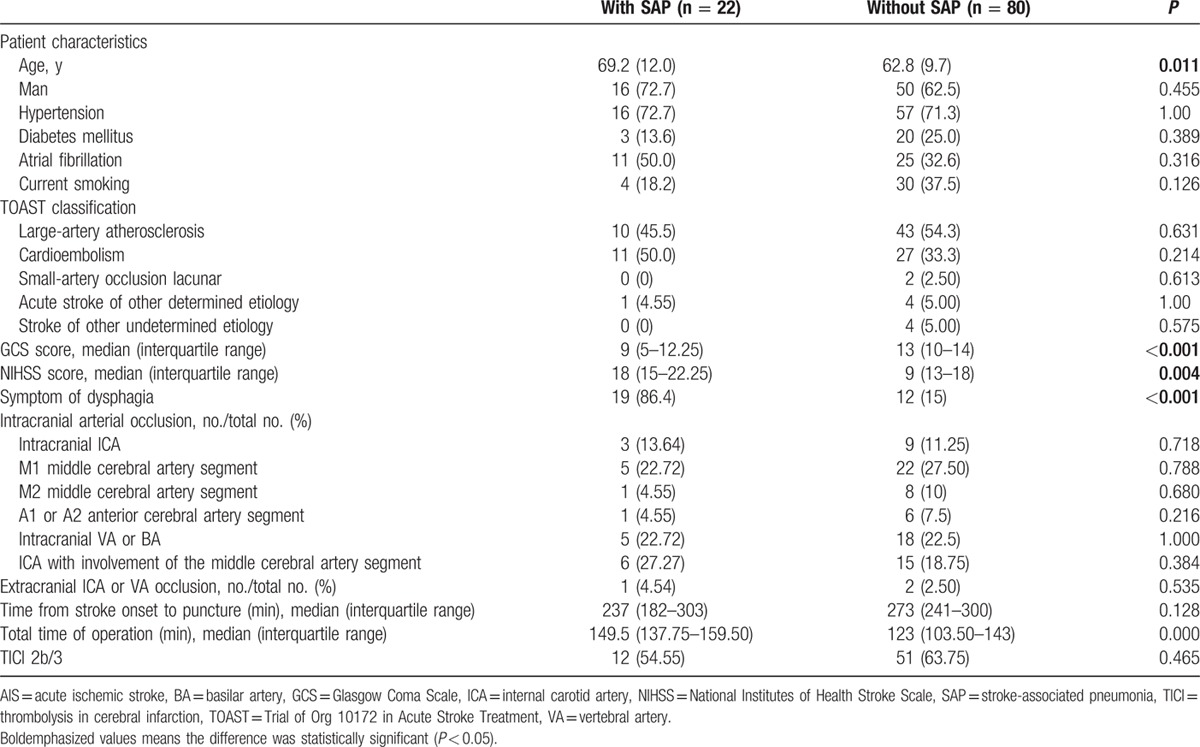
Univariate analysis of variables associated with in-hospital SAP after AIS.

**Table 2:**
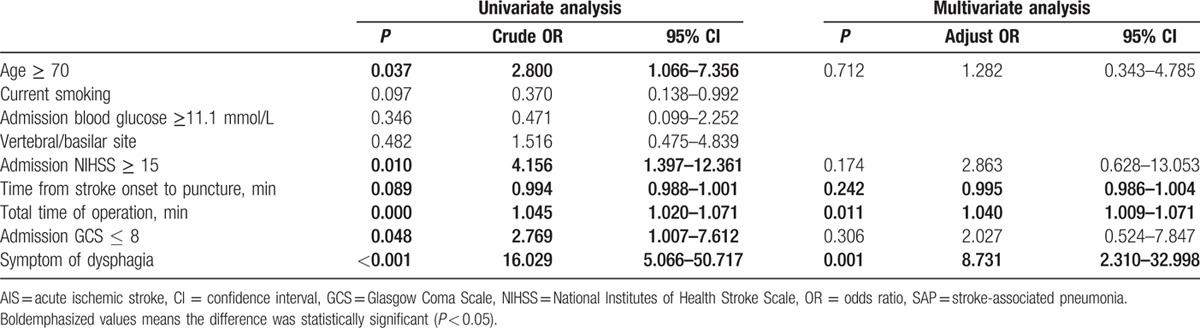
Multivariable analysis of parameters associated with in-hospital SAP after AIS.

### 3.3 Clinical outcomes

**Figure 3:**
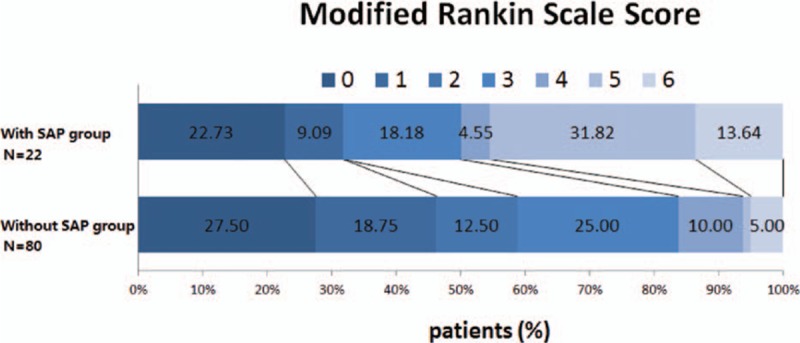
Modified Rankin scale scores at 90 d in the 2 groups. SAP = stroke-associated pneumonia.

**Figure 4:**
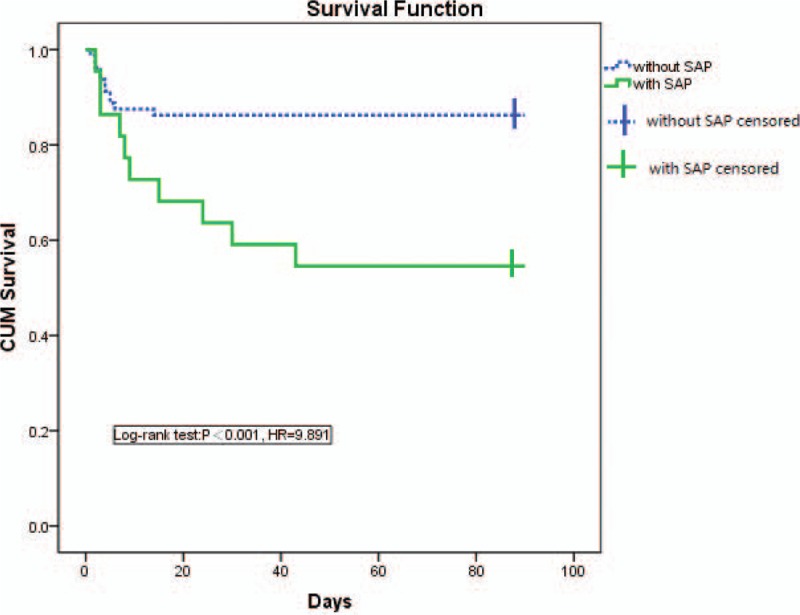
Kaplan–Meier survival curve depicting 90-d survival of patients with acute ischemic stroke underwent intra-arterial treatment. SAP = stroke-associated pneumonia.

Patients without SAP were more likely to occur a favorable outcome (mRS ≤ 2) than those with SAP (48.8% vs 31.8%, respectively; *P* = 0.031). Four of all 80 patients without SAP and 3 of all 22 patients with SAP died in hospital (5.0% vs 13.6%, respectively; *P* = 0.169). After 3 months, the mortality rates for patients without SAP were lower than those with SAP (8.75% vs 22.7%, respectively; *P* < 001). Patients with SAP had stable or improved deficits after AIS with IAT (*P* < 0.001) (Figs. 3 and 4).

## 4 Discussion

Our results show that age, NIHSS score, GCS score, duration time of operation, and symptom of dysphagia were significantly associated with SAP in patients with AIS with IAT within 6 h. The associations of age, NIHSS score, and GCS score with SAP were attenuated after adjusting for presence of dysphagia and total time of operation. This study also demonstrated that patients with AIS underwent IAT with SAP had a significantly poorer functional outcome and increased mortality rate.

With the publication of MR CLEAN, ESCAPE, EXTEND-IA, and SWIFT PRIME trials, IAT has been considered as a potently effective treatment for AIS. The studies suggest that IAT should be offered to patients who have documented occlusion in the anterior circulation. The rate of TICI 2b/3 is 61.76% in our study, and the difference between SAP group and no SAP group was no significant. In the previous IAT studies, the rate of TICI 2b/3 was reported >70%,^[1,25–28]^ which can be explained that the patients included in this study were different from those in the above studies. About 16.6% patients studied in this research were confirmed to be posterior circulation occlusion.

Several research has revealed that SAP is in connection with a higher rates of mortality and less favorable prognosis.^[29]^ In this study, the incidence of SAP was remarkably higher than in other stroke unit studies (21.6% vs 4–12%, respectively). One explanation might be in some of the comparable stroke unit studies, the neurologic deficit of patients was less severe than in our study (NIHSS score, 7–8, 9 vs 15, respectively).^[29–31]^ A second potential explanation may be our study including data from patients’ stay in the stroke unit and the peripheral ward, and prospective study reported that rate of SAP (19%) performed in even less severely affected patients (NIHSS score, 5) in a mixed setting.^[22]^

Several risk factors for SAP have been identified. Consistent with the prior studies, in-hospital SAP was significantly associated with older age, present history of atrial fibrillation, congestive heart failure, chronic obstructive pulmonary disease and smoking, prestroke dependence, admission NIHSS score, GCS score, dysphagia, blood glucose, and stroke subtypes.^[11–16]^ Some clinical score were developed and validated for predicting SAP after AIS, such as A^2^DS^2^ clinical score and 34-point AIS-APS.^[15,16]^ In our study, the SAP group had obvious older age, higher NIHSS score, lower GCS score, longer total time of operation, and higher rate of symptom of dysphagia. We also observed the tendency of higher rate of male sex and present history of atrial fibrillation in SAP group, but the difference was not significant compared with no SAP group. Because of the small sample size, the patients rolled in this study have not found any of congestive heart failure and chronic obstructive pulmonary disease. The most interesting result in this study is that only symptom of dysphagia and total time of operation were found identified as independent predictors of SAP in this study. One explanation of that is may be longer operation time coursing longer time to get reconstruction of brain blood flow, which may decrease the chances of favorable outcomes. After that older patients need more time for surgery because of the tortuous artery, and SAP is occurs more in older patients.

In our study, SAP patients’ deficits were stable or improved. Compared with SAP group, survival of SAP group was prolonged.^[22]^ In other studies, mortality evaluated at 30 days or at time of discharge from hospital was between 10.1% and 37.3%. Mortality evaluated after 12 months was reported to be between 49% and 60.1%.^[5,8,10,17]^ In this study, the Kaplan–Meier 90-day survival estimate (12.7%) was lower than MR CLEAN study (21%),^[1]^ but higher than ESCAPE study (10%).

Our study had some limitations. First, SAP is a complex disease, and various factors play—or are assumed to play—a role in pathogenesis. As an observational study, we cannot rule out the possibility that some nonincluded baseline variables might have some impact on the development of in-hospital SAP after AIS. In addition, our study is a small sample size study. Several confirmed risk factors for SAP have not been identified in this research. Second, as we only have information on new-onset SAP during hospitalization without documentation of the exact date, our data allow no conclusion as to whether patients with a longer length of stay to develop pneumonia or whether diagnosis of pneumonia leads to a longer hospitalization.

In conclusion, this study suggested that beside dysphagia, total time of operation is a risk factor of SAP in patients with AIS with IAT. SAP is associated with poor functional outcome and higher mortality after AIS underwent IAT. Our results reinforce the need for identifying patients with AIS underwent IAT at high risk for pneumonia.

## References

[1] Berkhemer OA Fransen PS Beumer D A randomized trial of intraarterial treatment for acute ischemic stroke. *N Engl J Med* 2015; 372:11–20. 25517348 10.1056/NEJMoa1411587

[2] Fuentes B Alonso DLM Ximenez-Carrillo A Futile interhospital transfer for endovascular treatment in acute ischemic stroke: the Madrid stroke network experience. *Stroke* 2015; 46:2156–2161. 26106117 10.1161/STROKEAHA.115.009282

[3] Powers WJ Derdeyn CP Biller J 2015 American Heart Association/American Stroke Association Focused Update of the 2013 Guidelines for the Early Management of Patients With Acute Ischemic Stroke Regarding Endovascular Treatment: A Guideline for Healthcare Professionals From the American Heart Association/American Stroke Association[J]. *Stroke* 2015; 46:3020–3035. 26123479 10.1161/STR.0000000000000074

[4] Katzan IL Dawson NV Thomas CL The cost of pneumonia after acute stroke. *Neurology* 2007; 68:1938–1943. 17536051 10.1212/01.wnl.0000263187.08969.45

[5] Koennecke HC Belz W Berfelde D Factors influencing in-hospital mortality and morbidity in patients treated on a stroke unit. *Neurology* 2011; 77:965–972. 21865573 10.1212/WNL.0b013e31822dc795

[6] Heuschmann PU Kolominsky-Rabas PL Misselwitz B Predictors of in-hospital mortality and attributable risks of death after ischemic stroke: the German Stroke Registers Study Group. *Arch Intern Med* 2004; 164:1761–1768. 15364669 10.1001/archinte.164.16.1761

[7] Kass-Hout T Kass-Hout O Sun CH Periprocedural cost-effectiveness analysis of mechanical thrombectomy for acute ischemic stroke in the stent retriever era. *Interv Neurol* 2015; 3:107–113. 26019714 10.1159/000371729 PMC4439777

[8] Lakshminarayan K Tsai AW Tong X Utility of dysphagia screening results in predicting poststroke pneumonia. *Stroke* 2010; 41:2849–2854. 20947835 10.1161/STROKEAHA.110.597039 PMC2994997

[9] Chamorro A Meisel A Planas AM The immunology of acute stroke. *Nat Rev Neurol* 2012; 8:401–410. 22664787 10.1038/nrneurol.2012.98

[10] Hannawi Y Hannawi B Rao CP Stroke-associated pneumonia: major advances and obstacles. *Cerebrovasc Dis* 2013; 35:430–443. 23735757 10.1159/000350199

[11] Kammersgaard LP Jorgensen HS Reith J Early infection and prognosis after acute stroke: the Copenhagen Stroke Study. *J Stroke Cerebrovasc Dis* 2001; 10:217–221. 17903827 10.1053/jscd.2001.30366

[12] Aslanyan S Weir CJ Diener HC Pneumonia and urinary tract infection after acute ischaemic stroke: a tertiary analysis of the GAIN International trial. *Eur J Neurol* 2004; 11:49–53. 14692888 10.1046/j.1468-1331.2003.00749.x

[13] Chumbler NR Williams LS Wells CK Derivation and validation of a clinical system for predicting pneumonia in acute stroke. *Neuroepidemiology* 2010; 34:193–199. 20197702 10.1159/000289350 PMC2883837

[14] Sellars C Bowie L Bagg J Risk factors for chest infection in acute stroke: a prospective cohort study. *Stroke* 2007; 38:2284–2291. 17569875 10.1161/STROKEAHA.106.478156

[15] Ji R Shen H Pan Y Novel risk score to predict pneumonia after acute ischemic stroke. *Stroke* 2013; 44:1303–1309. 23482598 10.1161/STROKEAHA.111.000598

[16] Hoffmann S Malzahn U Harms H Development of a clinical score (A^2^DS^2^) to predict pneumonia in acute ischemic stroke. *Stroke* 2012; 43:2617–2623. 22798325 10.1161/STROKEAHA.112.653055

[17] Vermeij FH Scholte ORW de Man P Stroke-associated infection is an independent risk factor for poor outcome after acute ischemic stroke: data from the Netherlands Stroke Survey. *Cerebrovasc Dis* 2009; 27:465–471. 19329851 10.1159/000210093

[18] Harms H Prass K Meisel C Preventive antibacterial therapy in acute ischemic stroke: a randomized controlled trial. *PLoS ONE* 2008; 3:e2158. 18478129 10.1371/journal.pone.0002158 PMC2373885

[19] Prass K Braun JS Dirnagl U Stroke propagates bacterial aspiration to pneumonia in a model of cerebral ischemia. *Stroke* 2006; 37:2607–2612. 16946159 10.1161/01.STR.0000240409.68739.2b

[20] Liu X Xu G Wu W Subtypes and one-year survival of first-ever stroke in Chinese patients: the Nanjing Stroke Registry. *Cerebrovasc Dis* 2006; 22:130–136. 16691021 10.1159/000093241

[21] Mayer SA Copeland D Bernardini GL Cost and outcome of mechanical ventilation for life-threatening stroke. *Stroke* 2000; 31:2346–2353. 11022062 10.1161/01.str.31.10.2346

[22] Bruening T Al-Khaled M Stroke-associated pneumonia in thrombolyzed patients: incidence and outcome. *J Stroke Cerebrovasc Dis* 2015; 24:1724–1729. 26051666 10.1016/j.jstrokecerebrovasdis.2015.03.045

[23] Hacke W Kaste M Fieschi C Randomised double-blind placebo-controlled trial of thrombolytic therapy with intravenous alteplase in acute ischaemic stroke (ECASS II). Second European-Australasian Acute Stroke Study Investigators. *Lancet* 1998; 352:1245–1251. 9788453 10.1016/s0140-6736(98)08020-9

[24] Fugate JE Klunder AM Kallmes DF What is meant by “TICI”? *AJNR Am J Neuroradiol* 2013; 34:1792–1797. 23578670 10.3174/ajnr.A3496 PMC7965642

[25] Goyal M Demchuk AM Menon BK Randomized assessment of rapid endovascular treatment of ischemic stroke. *N Engl J Med* 2015; 372:1019–1030. 25671798 10.1056/NEJMoa1414905

[26] Campbell BC Mitchell PJ Kleinig TJ Endovascular therapy for ischemic stroke with perfusion-imaging selection. *N Engl J Med* 2015; 372:1009–1018. 25671797 10.1056/NEJMoa1414792

[27] Saver JL Goyal M Bonafe A Stent-retriever thrombectomy after intravenous t-PA vs. t-PA alone in stroke. *N Engl J Med* 2015; 372:2285–2295. 25882376 10.1056/NEJMoa1415061

[28] Jovin TG Chamorro A Cobo E Thrombectomy within 8 hours after symptom onset in ischemic stroke. *N Engl J Med* 2015; 372:2296–2306. 25882510 10.1056/NEJMoa1503780

[29] Weimar C Roth MP Zillessen G Complications following acute ischemic stroke. *Eur Neurol* 2002; 48:133–140. 12373029 10.1159/000065512

[30] Ifejika-Jones NL Arun N Peng H The interaction of aspiration pneumonia with demographic and cerebrovascular disease risk factors is predictive of discharge level of care in acute stroke patient. *Am J Phys Med Rehabil* 2012; 91:141–147. 22355814 10.1097/phm.0b013e31823caa8d

[31] Minnerup J Wersching H Brokinkel B The impact of lesion location and lesion size on poststroke infection frequency. *J Neurol Neurosurg Psychiatry* 2010; 81:198–202. 19726403 10.1136/jnnp.2009.182394

